# Multilayer perceptron-genetic algorithm as a promising tool for modeling cultivation substrate of *Auricularia cornea* Native to Iran

**DOI:** 10.1371/journal.pone.0281982

**Published:** 2023-02-21

**Authors:** Akbar Jahedi, Mina Salehi, Ebrahim Mohammadi Goltapeh, Naser Safaie

**Affiliations:** 1 Department of Plant Pathology, Tarbiat Modares University, Jalal, Iran; 2 Department of Plant Genetics and Breeding, Tarbiat Modares University, Jalal, Iran; Indian Council of Agricultural Research-Directorate of Mushroom Research, INDIA

## Abstract

*Auricularia cornea* Ehrenb (syn. *A*. *polytricha*) is a wood-decaying fungi known as black ear mushroom. Earlike gelatinous fruiting body distinguishes them from other fungi. Industrial wastes have the potential to be used as the basic substrate to produce mushrooms. Therefore, 16 substrate formulations were prepared from different ratios of beech (BS) and hornbeam sawdust (HS) supplemented with wheat (WB) and rice brans (RB). The pH and initial moisture content of substrate mixtures were adjusted to 6.5 and 70%, respectively. The comparison of *in vitro* growth characteristics of the fungal mycelia under the different temperatures (25, 28, and 30°C), and culture media [yeast extract agar (YEA), potato extract agar (PEA), malt extract agar (MEA), and also HS and BS extract agar media supplemented with maltose, dextrose, and fructose revealed that the highest mycelial growth rate (MGR; 7.5 mm/day) belonged to HS and BS extract agar media supplemented with three mentioned sugar at 28°C. In *A*. *cornea* spawn study, the substrate combination of BS (70%) + WB (30%) at 28°C and moisture contents of 75% displayed the highest mean MGR (9.3 mm/day) and lowest spawn run period (9.0 days). In the bag test, “BS (70%) + WB (30%)” was the best substrate displaying the shortest spawn run period (19.7 days), and the highest fresh sporophore yield (131.7 g/bag), biological efficiency (53.1%) and number of basidiocarp (9.0/bag) of *A*. *cornea*. Also, *A*. *cornea* cultivation was processed to model yield, biological efficiency (BE), spawn run period (SRP), days for pinhead formation (DPHF), days for the first harvest (DFFH), and total cultivation period (TCP) by multilayer perceptron-genetic algorithm (MLP-GA). MLP-GA (0.81–0.99) exhibited a higher predictive ability than stepwise regression (0.06–0.58). The forecasted values of the output variables were in good accordance with their observed ones corroborating the good competency of established MLP-GA models. MLP-GA modeling exhibited a powerful tool for forecasting and thus selecting the optimal substrate for maximum *A*. *cornea* production.

## Introduction

*Auricularia* is a genus of medicinal mushroom known as edible jelly or wood ear mushrooms and accordingly classified as wood-decaying mushrooms. Growth of this fungus is oftentimes observed on decayed logs and dead branches of trees during the rainy season in the forests around the world [1, 2]. The distribution of *Auricularia* spp. have been reported in tropical and subtropical areas of the world [3, 4]. It is noteworthy that three mushroom genera viz., *Lentinula*, *Pleurotus*, and *Auricularia* accounted for 63% of the global mushroom production in 2019 [5]. *Pleurotus ostreatus* production has been marginally increased since 2010 and its rank dropped to third place in 2019. Shiitake and *Auricularia* spp. production has been significantly enhanced and their position upgraded from second and third in 2010 to first and second, respectively, in 2019 [6]. The nutritional and medicinal attributes of *Auricularia* spp. have resulted in increasing demand for the domestication of this mushroom [7]. The characteristics of *Auricularia*, basidiocarp of 3~8 cm (up to 12 cm) diameter, and noticeably ear-like form in brown, distinguish it from other fungi [8]. Moreover, the mentioned fungus has initially gelatinous and elastic properties and then gently becomes dry and fragile in the mature phase [9]. This mushroom is customarily appended to the substrate by a very short stalk and often lateral side. Also, the outward surface is mostly lidded with fluffy grey color and tiny hairs [10]. Nutritionally, the fruit body of this mushroom contains essential elements including minerals, polysaccharides, vitamins, and as well as over 30% of dry weight is a protein [7]. In addition to its importance as a food source, *Auricularia* spp. can be used for medicinal purposes to produce new therapeutic drugs for many human diseases [11]. The biologically active compounds of *Auricularia* spp. including polysaccharides showed antitumor, antiviral, antibacterial and antiparasitic and immune system modulation properties [12, 13].

*Auricularia* is the first mushroom domesticated for industrial purposes due to its nutritional and medicinal properties [7]. *Auricularia* is a group of native fungi found in the northern forest of Iran, and they can be introduced to the medicinal mushroom industry in Iran because of their potential. The cultivation area of *A*. *cornea* Ehrenb (synonym: *A*. *polytricha*) is persistently increasing because of high medicinal and economic value [14]. It is shown that fruit bodies of *A*. *polytricha* were brighter in the lower temperature (15.4°C) but larger in size at the higher temperature (44.3°C) [15]. A previous study [16] examined the sawdust of the hardwood species including *Shorea* sp., *Falcataria moluccana*, and *Tectona grandis* as basal cultivation substrate of *A*. *polytricha* mushroom. The fastest mycelial growth (5.7 mm/day) was recorded for *Shorea* sp. [16]. Generally, the cultivation period lasted 80 days, and the substrate made of *F*. *moluccana* produced the highest yield of fruiting bodies (65.4 g/bag), total dry weight (7.6 g/bag), and fruiting bodies (11/bag). One study [17] was examined the effect of *Mansonia altissima* sawdust substrate supplemented with additives including *Sorghum bicolor* chaff, oil palm fiber, corn chaff, brewer grains, and wheat bran (0, 5, 10 and 20%) on the biological efficiency of *A*. *auricula*. A 20% increase in all the additives enhanced the biological efficiency of *A*. *auricula* [17]. Furthermore, among five additives used in the substrate; wheat bran was introduced as the best case [17]. Knowledge about important properties of suitable substrate for mycelium growth and spawn run is necessary [18–20]. Numerous studies [19–21] have shown that sorghum and millet grain has successfully been applied for spawn preparation. A previous study [22] evaluated the most favorable conditions for mycelial growth and yield of *A*. *polytricha* in different culture media as well as grain spawn and fruiting substrates. The fastest mycelial ramification was observed in coconut water gelatin in terms of mycelial thickness and mycelial growth rate (MGR; 13.2 mm/day). On the other hand, sweet sorghum grains showed the highest MGR (16 mm/day). Also, the compound of lime, rice bran, and lumber sawdust had the fastest mycelial run (10 mm/day), the highest yield (254 g/kg), and biological efficiency (30.79%). Another experiment [23] studied *A*. *polytricha* cultivation in sawdust amended with 30, 45, and 60% of *Zea mays*, *Pennisetum purpureum*, and *Panicum repens* stalks, respectively. The results revealed that the best substrate for mycelial growth was sawdust amended with *P*. *purpureum* stalk with a total colonization period of 32 days [23]. Furthermore, the most suitable substrate for the biological efficiency was sawdust amended with 60% of *P*. *purpureum* stalk (148.12%) followed by 30% of *Z*. *mays* (145.05%), 45% of Z. mays (144.15%), and 30% of *P*. *repens* (136.68%) stalks [23]. Overall, the results showed that the use of *Z*. *mays* and *P*. *repens* stalks on partially replaced sawdust is feasible to cultivate *A*. *polytricha* [23]. Moreover, *A*. *polytricha* was cultured using the formulations of oil palm wastes mixed with sawdust [24]. The results showed that the best substrate formulations were sawdust mingled with an oil palm frond (90:10) supplemented with 15% spent grain displaying a MGR of 8 mm/day, and also sawdust mingled with an empty fruit bunch (50:50) plus 10% spent grain showing a MGR of 7 mm/day [24]. Furthermore, the highest total fresh yield (188.9 g/bed) was obtained on sawdust mixed with an oil palm frond (90:10) supplemented with 15% spent grain and 85% moisture content showing biological efficiency (BE) of 288.9% [24]. It was stated that wheat bran added to paddy straw (1:3) showed the minimum days for spawn run (21.3 days), pinhead formation (31.3 days), first harvest (35.6 days), and also the highest yield (147.6 g/bed), and biological efficiency (59.04%) for *A*. *polytricha* cultivation.

A large amount of beech (BS) and hornbeam sawdust (HS) is produced in the wood industry in Iran [25, 26]. This solid waste is an environmental concern [27], and its reuse is essential. In this study, BS and HS supplemented with wheat bran (WB) and rice bran (RB) was used as the substrate to assess the compatibility of the substrates obtained endemic existence industrial wastes for Iranian wood ear mushroom production. The substrate combination should be optimized to achieve the maximum yield of *A*. *cornea* cultivation. However, the substrate optimization for yield and biological efficiency (BE) enhancement, and also spawn run period (SRP), days for pinhead formation (DPHF), days for the first harvest (DFFH), and total cultivation period (TCP) decrement of *A*. *cornea* are time- and cost-consuming. Studying the relationship among input variables “concentration levels of substrate components (BS, HS, WB, and RB)” and output variables “yield, BE, SRP, DPHF, DFFH, and TCP of *A*. *cornea*” could facilitate the substrate optimization for the production of this valuable edible and medicinal mushroom. Multivariate statistical techniques including stepwise regression (SR) have been used in the biological data analyses [28–30]. There is no research to study SR for modeling and predicting yield, BE, SRP, DPHF, DFFH, and TCP of *A*. *cornea*. SR is a popular data-mining technique involving the selection of the explanatory variables from a group of input variables for building a model [31].

**Fig 1:**
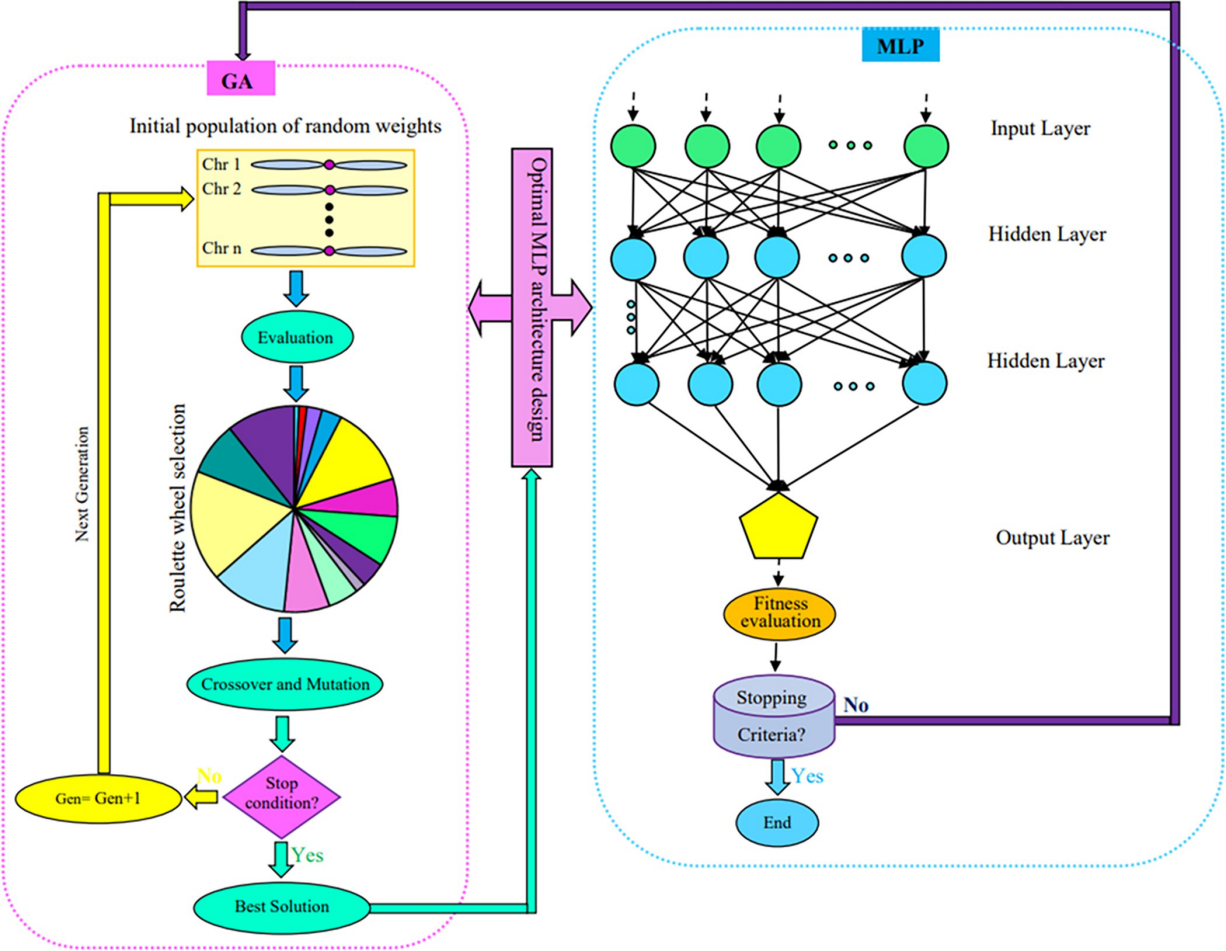
Schematic diagram of integrating multilayer perceptron (MLP) with genetics algorithm (GA) for MLP architecture optimization.

Artificial intelligence (AI) displays superior non-linear fitting and predictive ability to traditional modeling methods comprising regression models [29, 30, 32, 33]. Artificial neural networks (ANNs), likewise known as neural networks, are one subfield of AI connected with brain-inspired algorithms simulating human brain structure and function to find complicated and highly nonlinear relationships amongst factors (input) and parameters (output) variables [29, 32, 33]. Multilayer perceptron-genetic algorithm (MLP-GA; Fig 1) is one of the most popular hybrid learning algorithms displaying superb forecasting ability than traditional statistical techniques for computing the mathematical functions to analyze and decipher unpredictable data sets in plant and fungal research [32, 34, 35]).

The objectives of this research were (a) to optimize cultivation substrate for spawn and fruiting body production, (b) to approximate yield, BE, SRP, DPHF, DFFH, and TCP of *A*. *cornea* using regression technique “SR”, (c) to develop MLP-GA models to forecast output variables “yield, BE, SRP, DPHF, DFFH, and TCP of *A*. *cornea*” based on the concentration level of cultivation substrate components (input variables “HS, BS, RB and WB”), (d) to compare the performance of MLP-GA and regression method (SR) regarding prediction accuracy of output variables, and (e) to find the most important factors for maximum yield and BE, and also minimum SRP, DPHF, DFFH and TCP of *A*. *cornea*.

## Materials and methods

### Collection of mushroom isolates

The first stage in any mushroom production process is the preparation of the pure mycelial culture of the mushroom isolates (S1 Fig in S1 File). The isolates were collected from unprotected areas of Hyrcanian forests (36°053′11″N, 54°07′14″E and 36°020′54″N, 53°014′24″E) in the north of Iran with guidance of local authority.

### Pure culture of mushroom

The purification of isolates was performed by the method of Weber and Webster [36]. The tissue of the isolates was cultured on malt extract agar (MEA; 2%) and then incubated in a dark sterile space at 28°C for 5–8 days. The pure cultures were obtained by sub-culturing on fresh MEA and potato dextrose agar (PDA) media and then allowing the mycelium to fully colonize the Petri dishes. The best strain (AJ01) was determined according to some characters including MGR, density texture, and colony type.

### DNA extraction, amplification, sequencing and phylogenetic analyses

The best strain (AJ01) was cultured in PD broth at 28°C with constant shaking for 7 days. The fungal mycelia were freeze-dried and Genomic DNA was extracted with a procedure described by Salehi et al., [37, 38]. *ITS1-5*.*8S-ITS2* region was amplified with primers ITS1 and ITS4 [39] and *RPB2* using fRPB2-5F and fRPB2-7cR primers [40]. PCR reaction mixtures (25μl) consisted of 1μl genomic DNA (~100ng), 1μl forward and reverse primers (10 pM), and 12.5 μl Premix Taq (TaKaRa Biotechnology Ltd., Japan), and 10.5μl PCR quality water. PCR reaction programs were an initial denaturation at 94°C for 3 min, followed by 30 cycles of denaturation (94°C for 30 s), annealing (55°C (ITS) and 57°C (RPB2) for 30 s), extension (72°C for 1min) and a final extension at 72°C for 5 min. The PCR products were analyzed by agarose gel electrophoresis and purified using a DNA gel extraction kit (Axygen Biotechnology Ltd., China). The purified PCR product was directly sequenced using the same primers by Biomagic Company, China, and manually adjusted with chromas software (www.technelysium.com.au/chromas.html).

Basic Local Alignment Search Tool (BLAST; https://blast.ncbi.nlm.nih.gov/Blast.cgi) was carried to compare new *ITS* and *RPB2* sequences with other sequences (S1 Table in S1 File) accessible in NCBI database. The datasets of combined sequences of *ITS* and *RPB2* were separately aligned using the Q-INS-i algorithm of an online version of MAFFT v.7.205 (https://mafft.cbrc.jp/alignment/server/) [41]. The Gblocks program (version 0.91b) with all the three less stringent parameters (Allow smaller final blocks, Allow gap positions within the final blocks, and Allow gap positions within the final blocks) was utilized for editing the alignments. The best model for combined sequences of *ITS* and *RPB2* was chosen using PAUP/MrModeltest.2 [42]. Bayesian analyses were carried out using MrBayes 3.1.2 [43] with the general time-reversible model, including a gamma distribution for rates across sites and a proportion of invariant sites (GTR+I+G) model for ITS-RPB2 with three million generations. Dendroscope V.3.2.8 was used for the visualization of output files prepared by phylogenetic programs [44].

### Mycelial growth measurement

As MGR for colonizing the substrate is one of the important factors in mushroom cultivation. Therefore, in the first step, MGR was investigated in the different culture media including yeast extract agar (YEA), potato extract agar (PEA), malt extract agar (MEA), HS and BS extract agar media mixed with dextrose, fructose, and maltose as carbon sources at three different temperatures (25, 28, and 30°C). The one agar plug (5 mm diameter) from the stock culture was placed on the center of the surface of Petri dishes and incubated at mentioned temperatures in dark to fully colonize the plate. The MGR of *A*. *cornea* was determined by measuring the diameter of the colony in four directions. This experiment was done with four replications. This experiment was designed as factorial on Completely randomized design (CRD) with four replications.

### Preparation of sawdust medium for spawn production

The sawdust of two hardwood trees including BS and HS was used for spawn production. WB, RB, and CF are also considered as supplements. To carry out this stage of mushroom production, the different ratios of the sawdust to supplement [70:30, 100:0, and 0:100 (w/w)] were manually mixed. Then water [65, 75, and 90% (v/w)] was added to the mixture of sawdust and supplements after passing off it through a sieve with a pore size of 5 mm diameter. Final moisture content of the substrates was measured by weighing out a small sample of substrates, drying it in an oven, weighing it again and calculating moisture content (%). The medium pH was adjusted to 6.5 with CaCO3 (1%) for the spawn production. Finally, 100 g of each spawn formulization were put in 200 ml heat-resistant glass bottles and sterilized at 121°C under 1.4 atmospheric pressure for 60 min. The pure mycelium was obtained from the previous step for spawn production. One mycelial agar plug (1 cm diameter) of 4-day-old culture was placed on the center of prepared medium surface and incubated at three temperatures (25, 28, and 30°C) to completely colonize the culture medium. MGR was measured in four directions of glass bottles at 3-day intervals after cultivation with five replicates. This experiment was set up on a randomized complete block design (RCBD) with four replications.

### Fruiting body production

Sixteen substrate formulations were prepared from the sawdust of two hardwood trees (BS and HS) mixed with WB and RB in different ratios (S2 Table in S1 File) with a moisture content of 70%. The PH of the substrate mixture was adjusted to 6.5 by adding CaCO_3_. The substrates mixtures (825 g) were packed into the autoclavable polyethylene bags (40 cm in height and 15 cm in diameter). The top of the bags was tightly closed by using sterile cotton strings and sterilized at 121°C under 1.4 atmospheric pressure for 90 min. The bags were inoculated with sawdust spawns (10 g) taken from the previous stage and incubated in dark at 28 ± 1°C, then allowed to fully colonize the substrates. After the colonization stage was completed, the bags were transferred to the mushroom production room, then the top of the bags were removed while slitting by a sharp knife in four vertical directions. The primordial formation was induced by exposing the bags to temperature shock (15–20°C) for three days to stimulate fruiting body production. Air relatively humidity was maintained at 85–90% using flooding the floor and fogging system, and light exposure at 2000 lux set on a 12 h on/off cycle. The yield, number of fruiting body (NFB), BE [Eq (1)], SRP, DPHF, DFFH, and TCP were recorded for each substrate. This experiment was planned on a RCBD with 12 replicates to assess how substrates (S2 Table in S1 File) affected yield, NFB, BE, SRP, DPHF, DFFH, and TCP.


BE(%)=[(weightoffreshfruitingbody(g)/dryweightofusedsubstrate(g)]×100 (1)

### Carbon to nitrogen (C/N) analysis in experimental substrates

Carbon to nitrogen ratios (C/N) is one of the most important parameters that must be considered in the evaluation and cultivation of mushrooms. In this study, C/N was measured for spawn and fruiting body production tests [36, 45].

### Model development

Normalization of the data set was performed by Box-Cox transformation [46] before the machine learning algorithm test. Principal component analysis (PCA) was likewise used for finding outliers; but, no outliers were observed in the data set. The performance of all the tested models was evaluated using a five-fold cross-validation method with ten replicates. Accordingly, the best predictive model was found on unseen data from the entire data set. Stepwise regression (SR) and multilayer perceptron-genetic algorithm (MLP-GA) models were developed using the training data subset, and the predictive accuracy of the developed models was tested using the testing subset [47].

### Multilayer perceptron-genetic algorithm (MLP-GA) model

MLP-GA modeling (Fig 1) was applied to define the effects of BS, HS, WB, and RB concentration levels on yield, BE, SRP, DPHF, DFFH, and TCP of *A*. *cornea*. Three-layered feed forward back-propagation neural network was applied for learning and mapping the relationships between inputs (BS, HS, WB, and RB concentration levels) and outputs (yield, BE, SRP, DPHF, DFFH, and TCP of *A*. *cornea*). Hyperbolic tangent sigmoid (tansig) and pure lineral (purelin) transfer functions were used for hidden and output layers, respectively.

ANN predictive accuracy is influenced by its architecture, thus, evolutionary algorithms including GA are applied to design the optimal architecture of ANN [48]. The more and less number of hidden neurons than the problem complexity leads to overfitting and underfitting the data, and finally, reduces model performance. Underfitting causes a high error rate on both training and testing (unseen) subsets. Overfitting leads to high error rates on unseen data [49]. GA was applied to establish the fittest MLP structure (Fig 1). An initial population of 50, crossover rate of 0.85, generation number of 500, and mutation rate of 0.01 [32, 50, 51] were fixed for designing optimal MLP architecture. The performance of hybrid MLP-GA models was evaluated by the statistical criteria “coefficient of determination (R^2^) and root mean square error (RMSE) [29, 30, 32, 33].

### Sensitivity analysis of the models

Sensitivity analysis of MLP-GA model outputs was performed to find out the importance degree of the model inputs (BS, HS, WB, and RB concentration levels) on its outputs (yield, BE, SRP, DPHF, DFFH, and TCP of *A*. *cornea*). The sensitivity of yield, biological efficiency, spawn run, pinhead formation, first harvest, and total cultivation period of *A*. *cornea* was measured by variable sensitivity error (VSE) value representing MLP-GA model performance (RMSE) when that specific input variable is nonexistent in the model. The variable sensitivity ratio (VSR) value was computed as the ratio of VSE and MLP-GA model error (RMSE) when all input variables are present in the model. The input displaying higher VSR was recognized as a higher important input variable in MLP-GA model [28, 32, 33]. To make calculated VSRs more easily comparable, they were rescaled within the range [0, 1]. MATLAB [52] software was used to write the mathematical codes for developing and evaluating MLP-GA models.

## Results and discussion

### Phylogenetic analysis

**Fig 2:**
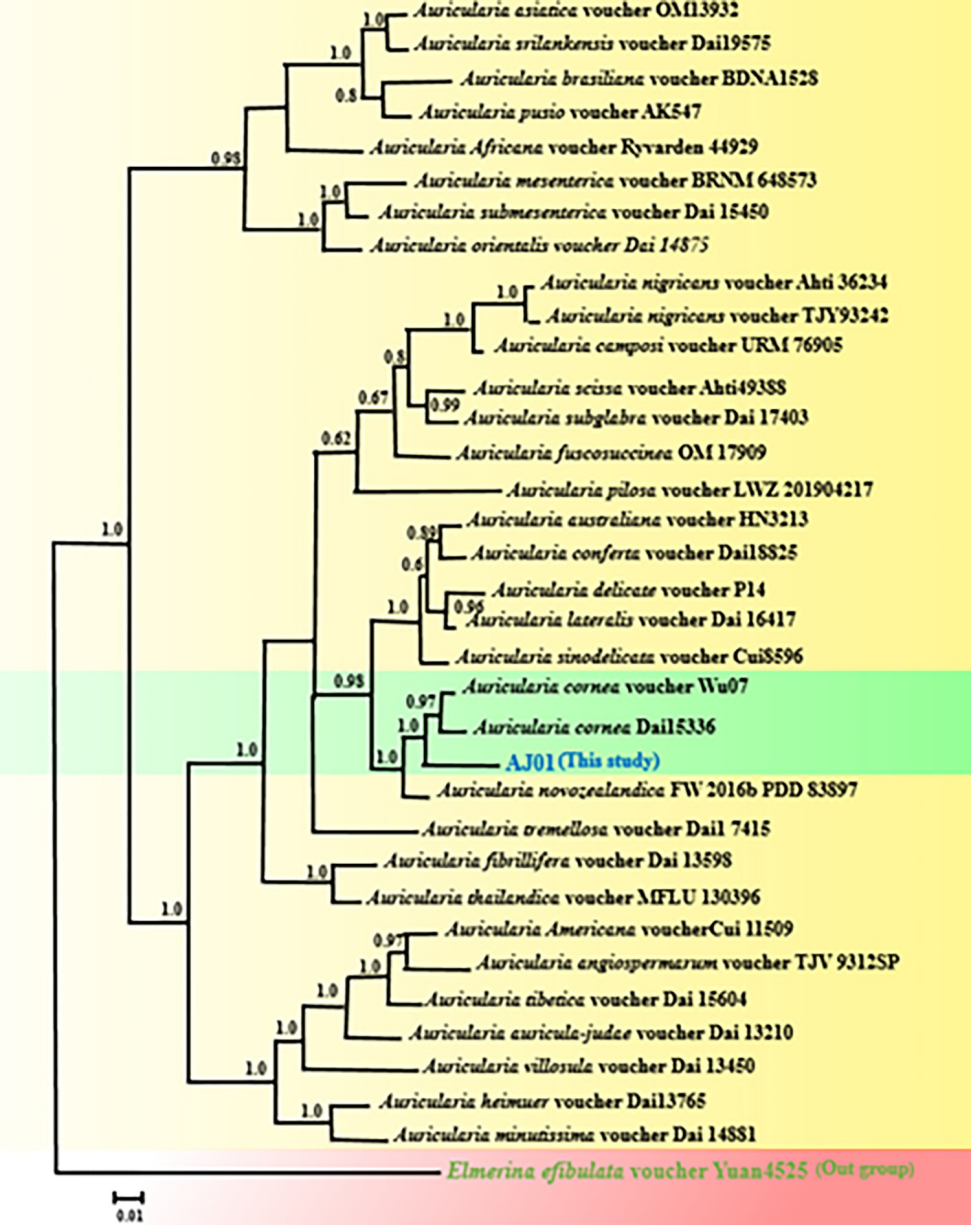
Bayesian tree illustrating the phylogeny of *Auricularia* species under GTR + G + I model based on the combined sequences of ITS1-5.8S-ITS2 region and RPB2 gene for *Auricularia cornea* (AJ01) in this study (bold font in the green area) and related species of this genus (in the yellow area), *Elmerina efibulata* voucher Yuan4525 was considered as outgroup. The sequences were aligned using MAFFT software and edited using Gblocks program. Bayesian posterior probabilities higher than 0.50 are given for appropriate clades.

By analysis of the sequences of *ITS1-5*.*8S-ITS2* region and *RPB2* gene, AJ01 was identified as *A*. *cornea* (syn. *A*. *polytricha*; Fig 2). The partial sequences of *ITS rDNA* and *RPB2* gene obtained from strain AJ01 were deposited in GenBank (NCBI) under the accession numbers OQ073696 and OQ107064, respectively.

### Effects of various media, temperatures, and carbon sources on mycelial growth in petri dish test

The different temperatures, sugars, and substrates significantly (p<0.01) affected MGR, growth period, morphology and growth type. As shown in Table 1 and S2 Fig in S1 File, HS (7.5 mm/day and 5.6 days) and BS (6.3 mm/day and 6.6 days) extract agar media with all three carbon sources “fructose, dextrose and maltose” and the temperature of 28°C displayed the highest MGR and accordingly the shortest period to fully colonize the Petri dish. In contrast, the lowest MGR (2.8 mm/day) was recorded at 25 and 30°C on YEA medium with dextrose as a carbon source. Also, the lowest MGR and consequently the longest period of mycelia completely colonizing Petri dish were observed on PEA, MEA and YEA media containing fructose, dextrose and maltose at 25, 28 and 30°C as well as HS agar extract medium containing fructose and dextrose at 25°C and BS extract agar medium containing fructose, dextrose and maltose at 25°C (Table 1). It was demonstrated that *A*. *polytricha* cultured in coconut water gelatin displayed the higher mycelial growth as compared to that in potato sucrose gelatin [22]. A previous study [53] reported that the different media showed significant difference in regard to *A*. *polytricha* mycelial growth. Carrot extract agar (CEA; 9.0 cm) was found to be the best media, the next best media were oat meal agar (OMA; 8.9 cm), and malt extract agar (MEA; 8.5 cm) which were significantly superior to PDA (7.7 cm). Also, *A*. *polytricha* displayed the highest mycelial growth (8.9 cm) at 30°C, followed by 25°C (8.4 cm) and 20°C (8.2 cm) [53].

**Table 1:** Mycelial growth rate (MGR; mm day^-1^) and days for *Auricularia cornea* fully colonize the Petri dishes on potato extract agar (PEA), malt extract agar (MEA), yeast extract agar (YEA), hornbeam extract agar (HSA), beech extract agar (BSA) with different carbon sources “fructose, dextrose and maltose” at 25, 28 and 30°C.

Media	Sugar source	MGR			Days to fully colonize the Petri dishes			Linear growth
		25°C	28°C	30°C	25°C	28°C	30 oC	
**PEA**	**Fructose**	3.2±0.23de	3.8±0.33cde	3.2±0.23de	13.0±0.88abc	10.6±0.11bcd	13.1±0.88abc	+
	**Dextrose**	3.2±0.20de	3.8±0.23cde	3.2±0.20de	13.0±0.81abc	10.7±0.08bcd	12.9±0.80abc	++
	**Maltose**	3.2±0.17de	3.8±0.23cde	3.2±0.20de	13.0±0.88abc	10.8±066abc	12.9±0.80abc	+
**MEA**	**Fructose**	3.0±0.41de	3.2±1.30de	3.0±0.67de	13.6±0.78ab	12.8±0.80abc	13.5±0.35ab	++
	**Dextrose**	2.8±0.41e	3.2±0.90de	3.0±0.51de	14.6±0.88a	13.0±0.80abc	13.6±0.88b	+++
	**Maltose**	3.0±0.23de	3.2±0.33de	3.0±0.23de	13.5±0.88ab	12.8±0.28abc	13.7±0.19ab	++
**YEA**	**Fructose**	2.8±0.17e	3.2±0.23de	3.0±0.20de	14.6±0.80a	12.9±0.32abc	13.5±0.88ab	+
	**Dextrose**	2.8±0.17e	3.2±0.23de	2.8±0.17e	14.6±0.78a	12.9±0.90abc	14.6±0.09a	+
	**Maltose**	3.0±0.41 de	3.3±1.30de	3.0±0.67de	13.4±0.88ab	13.0±0.22abc	13.7±0.65ab	+
**HSA**	**Fructose**	4.2±0.33cde	**7.5±0.90a**	5.4±0.51bc	9.6±0.68cd	5.6±0.88f	7.7±0.88def	+
	**Dextrose**	4.2±0.23cde	**7.5±0.33a**	5.4±0.23bc	9.8±0.08cd	5.6±0.88f	7.8±0.66def	+
	**Maltose**	4.7±0.20bcd	**7.5±0.23a**	5.4±0.20bc	8.6±0.76de	5.7±0.88f	7.9±0.43def	++
**BSA**	**Fructose**	4.2±0.20cde	**6.2±0.25ab**	4.7±0.20bcd	9.6±0.88cd	6.6±0.88ef	8.7±0.41de	+
	**Dextrose**	3.9±0.51cde	**6.4±1.30ab**	4.7±0.67bcd	10.6±0.56bcd	6.7±0.88ef	8.7±0.88de	+++
	**Maltose**	4.2±0.41cde	**6.2±0.90ab**	4.7±0.51bcd	10.0±0.44cd	6.7±0.88ef	8.8±0.28de	+

Linear growth; +: The lowest degree, ++: The mediocre degree, +++: The highest degree. Average values (four replicates) ± standard error are given. Means followed by the same letter are not significantly different according to LSD at 0.01 probability level.

Knowledge about the characteristics of the mushroom mycelium such as MGR, type of mycelium, and growth temperature is essential for wild mushroom cultivation [18–20]. The linear growth and high-density mycelium was recorded on PEA, MEA and BSA with dextrose as a carbon source (Table 1). The hardwood tree sawdust extract agar media displayed the fastest MGR and a short period for colonization of the culture medium (Table 1). The culture media containing YEA are used as a suitable organic source for MGR [54]. In this study, the favorable temperature of 28°C ± 1°C was favorable for all treatments regarding MGR, however, no difference was observed between different temperature for some treatments (Table 1).

### Spawn studies

The most important part of mushroom production is spawn quality [55]. According to previous research [56], the ability of mushroom for substrate colonization depends on suitable substrate, and the selection of the supplements such as RB leading to stimulate spawn. The substrate combination of BS (70%) + WB (30%) with moisture content of 75% at 28°C displayed the highest MGR (9.3 mm/day; Table 2). A study [21] reported that adding bran as a supplement to the medium provides a protein element that can enhance MGR (two-folds). Accordingly, grains without supplement resulted in the lowest MGR as observed in 100% millet and millet/sorghum at a 1:1 ratio.

**Table 2:** Mycelia growth rate (MGR; mm day^-1^) and spawn run period (SRP) of *Auricularia cornea* on different substrates obtained from different concentration levels (%) of beech (BS) and hornbeam sawdust (HS), corn flour (CF), wheat (WB) and rice brans (RB) at 25, 28 and 30°C and moisture contents of 65, 75 and 90%.

Proportion of substrate components (%)	C/N (%)	25°C and 65%		28°C and 75%		30°C and 90%	
		MGR	SRP	MGR	SRP	MGR	SRP
HS (100)	111.9	2.3±0.63g	21.8±0.85a	3.0±0.82defg	19.0±1.08ab	3.3±0.63cdefg	18.3±0.85abc
BS (100)	106.4	3.3±0.63cdefg	18.8±0.85abc	4.0±0.82cdefg	17.0±1.08abc	2.5±0.50fg	21.5±0.65a
HS (70) + RB (30)	46.9	3.3±0.25cdefg	18.8±0.85abc	2.5±0.63cdefg	15.8±0.85cde	2.8±0.63efg	19.8±0.85ab
BS (70) + RB (30)	47.2	4.3±0.63cdefg	15.8±0.85cde	5.0±0.82cd	13.0±1.08efg	4.5±0.50cdefg	15.8±0.85cde
HS (70) + WB (30)	49.1	3.3±0.63cdefg	19.0±1.08ab	5.0±0.41cd	12.0±1.08gh	4.0±0.41cdefg	16.8±0.85bcd
BS (70) + WB(30)	49.2	5.0±0.41cd	13.0±1.80efg	9.3±0.63a	9.0±1.08h	5.3±0.63c	12.0±1.08fgh
HS(70) + CF(30)	64.9	2.3±0.63g	21.8±0.85a	5.3±0.63c	12.0±1.08fgh	3.5±0.5cdefg	18.8±0.85abc
BS(70) + CF(30)	64.1	4.3±0.25cdefg	16.8±0.85bcd	8.5±0.50b	11.0±1.08gh	4.8±0.75cde	15.0±1.08def

Average values (five replicates) ± standard error are given. Means followed by the same letter are not significantly different according to LSD at 0.01 probability level.

It is stated that the high rate of colonization could be attributed to the most appropriate proportion of substrate components with high storage of energy and the nutritional components such as nitrogen, carbon, lipids, and minerals [57]. Overall, the most appropriate temperature (28°C), moisture content (75%), and BS extract agar media supplemented with WB resulted in the highest MGR (Table 2). It can be supported by the study of [9] that showed WB increased MGR due to their protein-rich ingredients.

### Fruiting body substrate test

**Fig 3:**
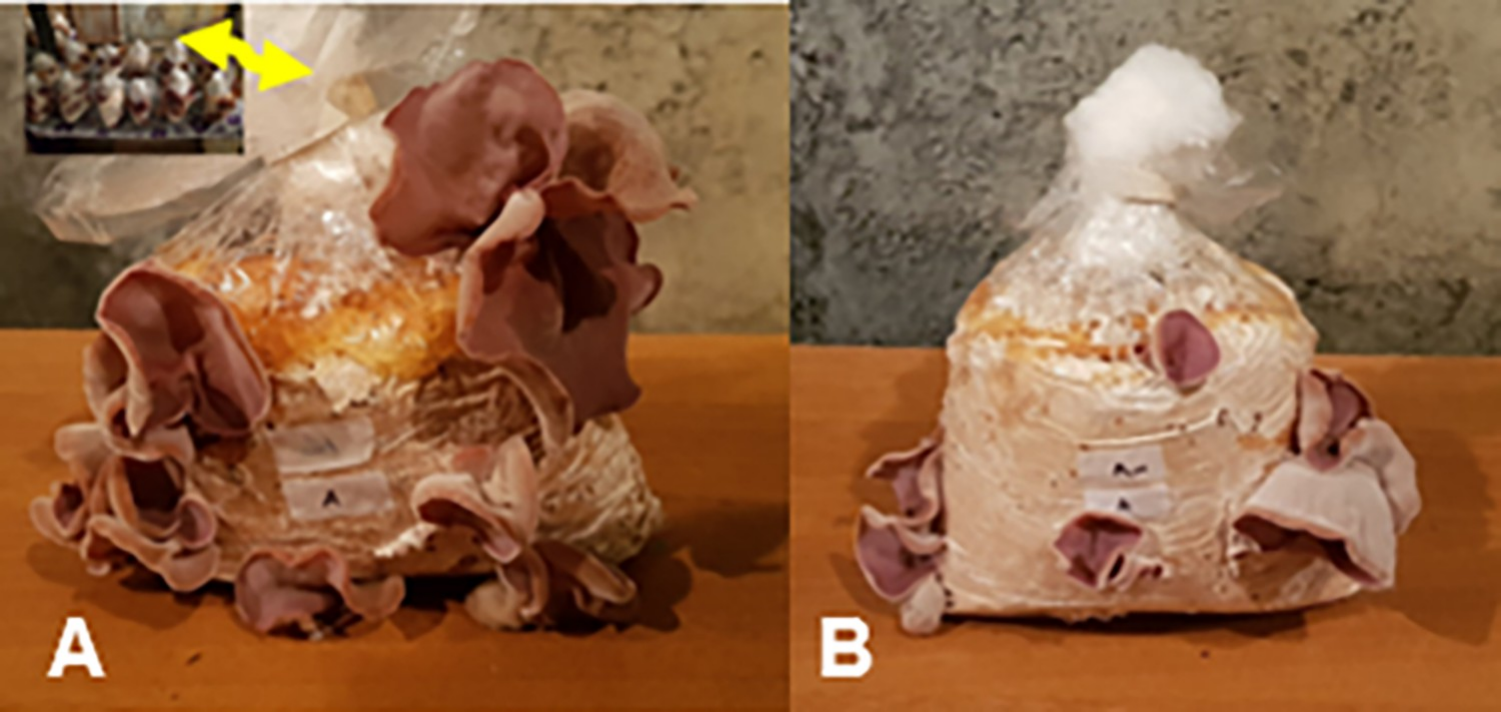
*Auricularia cornea* cultivation in “70% beech sawdust + 30% wheat bran” and “100% wheat bran” with moisture content of 70%, and pH = 6.5.

The results of an initial assessment revealed that the spawn run period was 19.7 to 41.5 days and additionally, the total period lasted, on average, 43.5 to 60.5 days in bags. For this research, the fruit body of *A*. *cornea* was obtained between 30.0 to 51.5 days after the first harvest. The results including SRP, DFPF, DFFH, NFB, yield, BE and TCP for each substrate are shown in Table 3. The different stages of *A*. *cornea* fruit body development are presented in S3 Fig in S1 File. In this study, the use of supplements as nutritional additives had a great effect on fruit body production (Table 3), so it can be explained by MGR increment in formulations supplemented with nutritional additives. In this research, BS (70%) + WB (30%) was introduced as the best substrate owing to the shortest SRP (19.7 days), the highest yield of fresh fruit body weight (131.7 g/bag), BE (53.1%), NFB (9/bag) and TCP (43.5 days) (Table 3; Fig 3). According to the previous studies [58, 59], spent grain provides a nitrogen source for mycelial growth in the substrate. However, the previous research [16] reported the shortest SRP (4 days) on the substrate made of *Shorea* sp. sawdust and the highest yield of fruiting bodies (65.4 g) on substrate obtained from *F*. *moluccana* sawdust. Based on the present study, the suitable performance of WB can be attributed to its high protein content which increases the nitrogen level of the substrate. The comparison of growth characteristics of the fungi growing on the different substrate conditions revealed the rate of 21.7 days for SRP of “BS (80%)+WB (20%)” and “BS (70%)+RB (30%)”. However, “BS (70%)+RB (30%)” displayed the higher yield as compared to “BS (80%)+WB (20%)” (Table 3). A previous study [60] reported that the use of different compounds with a lingo-cellulosic source (equal proportions of rice and *Leonotis* straws) for substrate formulation resulted in biological efficiency increment of *P*. *ostreatus*. The results of the present study were also consistent with the findings of another study [17] reporting the addition of wheat bran to the substrate increased the efficiency of the *A*. *auricula* mushroom. It was reported that mycelium growth is dependent on the quality of wheat bran’s nutrients [61]. The previous study [62] demonstrated that the substances including proteins, transaminase enzymes, and amino acids in wheat bran enhanced mycelium growth. It was also stated that the woody substrates containing high glucosamine increased fruiting body yield in *Pleurotus* spp. cultivation. Thus, fruiting body yield could be evaluated by the amount of mycelium grown during the cultivation period. Also, it was indicated that “RB + WB: paddy straw (3:1)” induced the faster mycelial growth of *A*. *polytricha* [63]. In the present study, the longest SRP was recorded for non-supplements HS substrate (41.5 days) that was significantly different from non-supplements BS (37.7 days) (Table 3). Between two sawdust without supplements, BS showed the higher yield and lower DFFH than HS while both sawdust (BS and HS) displayed no difference in DFPF, NFB, BE and TCP (Table 3).

**Table 3:** Effects of different substrate obtained from different concentration levels (%) of beech (BS) and hornbeam sawdust (HS) wheat (WB) and rice brans (RB) on yield, biological efficiency (BE), spawn run period (SRP), days for pinhead formation (DPHF), days for the first harvest (DFFH), number of fruiting body (NFB) and total cultivation period (TCP) of *Auricularia cornea*.

Proportion of substrate components	C/N (%)	SRP	DPHF	DFFH	NFB	Yield (g/bag)	BE (%)	TCP
HS(100)	111.9	41.5±0.65a	46.2±0.85a	51.5±0.65a	5.9±0.65cde	68.7±0.25j	27.9±0.25^g^	60.5±0.90a
BS(100)	106.4	37.7±0.48b	42.5±0.65a	49.5±0.65b	5.5±0.55de	71.7±0.65i	29.1±0.65^g^	60.5±0.93a
HS(90)+RB(10)	77.0	31.7±0.48d	36.7±0.48b	41.5±0.65d	6.3±0.65bcde	87.7±0.85h	35.5±0.85^f^	53.5±0.53b
BS(90) + RB(10)	76.4	27.7±0.48f	33.7±0.48bcd	39.5±0.65ef	7.1±0.80abcd	90.7±0.85g	36.7±0.85^f^	51.5±0.93bc
HS (90)+ WB(10)	111.3	25.7±0.17f	34.0±0.41bcd	38.5±0.65ef	7.2±0.45abcd	88.7±0.85gh	35.9±0.85^f^	50.5±0.46bcd
BS(90) + WB(10)	78.4	25.7±0.48g	32.2±0.48bcde	37.5±0.65f	7.5±0.66abcd	99.7±0.90f	40.3±0.90^e^	49.0±0.90cde
HS(80) + RB(20)	58.2	29.7±0.48e	35.7±0.85bc	40.5±0.65ed	7.4±0.65abcd	111.7±0.85e	45.1±0.85^d^	52.5±0.33bc
BS(80) + RB(20)	58.3	23.7±0.48h	29.2±1.31ed	38.5±0.65ef	7.6±0.65abcd	114.7±0.85d	46.3±0.85c^d^	50.5±0.99bcd
HS(80) + WB(20)	60.5	24.7±0.48hg	31.2±1.31cde	38.5±0.65ef	7.8±0.09abc	114.7±0.05d	46.3±0.05^cd^	50.5±0.75bcd
BS(80) + WB(20)	60.5	21.7±0.09i	28.5±1.55e	35.5±0.65g	8.2±0.60ab	117.7±0.16c	47.5±0.16^bcd^	45.5±0.43fg
HS(70) + RB(30)	46.9	27.7±0.21f	34.0±2.04bcd	38.5±0.65ef	8.2±0.65ab	116.7±0.80cd	47.1±0.80^bcd^	51.5±0.93bc
BS(70) + RB(30)	47.2	21.7±0.08i	29.7±1.80de	33.5±0.65g	8.8.6±0.71a	122.7±0.55b	49.5±0.55^b^	46.5±0.93efg
HS(70) + WB(30)	49.1	23.7±0.48h	31.0±2.29cde	34.5±0.65g	8.7±0.65a	120.7±0.85b	48.7±0.85^bc^	47.5±0.88def
BS (70)+ WB(30)	49.2	19.7±0.28j	27.5±2.53e	30.5±0.65h	9.0±0.65a	131.7±0.85a	53.1±0.85^a^	43.5±0.93g
WB(100)	18.0	37.7±0.38b	47.2±2.29a	50.5±0.65ab	4.5±0.10e	56.7±0.15l	23.1±0.15^h^	60.5±0.93a
RB(100)	17.7	34.7±0.48c	43.7±2.78a	47.5±0.65c	4.8±0.62e	61.7±0.66k	25.1±0.66^h^	59.5±0.33a

Average values (12 replicates) ± standard error are given. Means followed by the same letter are not significantly different according to LSD at 0.01 probability level.

### C/N analysis

As shown in Table 3, the different substrates significantly affected all the measured parameters. The sawdust substrates alone (HS and BS) with high C/N (111.9 and 106.4, respectively for HS and BS), and bran substrates alone (RB and WB) with low C/N (17.7 and 18, respectively for RN and WB) displayed the low yield and long SRP. The best condition for the maximum yield and minimum SPR of *A*. *cornea* was in the substrates that were firstly: made of two components, bran and sawdust and secondly, they had a C/N of about 49%. The best substrate displaying the highest yield and shortest period of the spawn run was BS (70) + WB (30) with 131.7 g/bag and 19.7 21.7 days of yield and SRP, respectively. The sawdust substrates (HS and BS) and bran substrates (RB and WB) alone had the longest TCP but the sawdust substrates showed a higher BE and yield as compared to bran substrates (Table 3). Although C/N is one of the most important parameters in the cultivation of mushrooms but the mushroom yield depended on the factors, other than C/N ratio, including the type and chemical and physical structure of compounds, vitamins, minerals, and other nutrients of substrate [64, 65].

### Regression analysis

The fit of stepwise regression models (exhibited by R^2^) for predicting yield (0.151), BE (0.478), SRP (0.114), DPHF (0.270), DFFH (0.511), and TCP (0.578) of *A*. *cornea* (Table 4) for testing subset showed that these developed SR models can describe 15.1, 47.8, 11.4, 27.0, 51.1 and 57.8% variability in yield, BE, SRP, DPHF, DFFH, and TCP of *A*. *cornea*, respectively, when they face data not used in training (Table 4).

**Table 4:** Statistics on stepwise regression (SR) and multilayer perceptron-genetic algorithm (MLP-GA) models for yield, biological efficiency (BE), spawn run period (SRP), days for pinhead formation (DPHF), days for the first harvest (DFFH), and total cultivation period (TCP) in *Auricularia cornea* cultivation on different substrates obtained from different ratios of beech and hornbeam sawdust, and wheat and rice brans.

	Models	Training subsets		Testing subsets	
		R^2^	RMSE	R^2^	RMSE
**SRP**	**SR**	0.13	6.013	0.114	6.263
	**MLP-GA**	**0.9844**	**0.7881**	**0.9691**	**1.206**
**DPHF**	**SR**	0.21	6.427	0.27	5.568
	**MLP-GA**	**0.81**	**3.026**	**0.82**	**2.594**
**DFFH**	**SR**	0.064	5.376	0.5114	5.705
	**MLP-GA**	**0.9654**	**1.01**	**0.9571**	**1.6628**
**Yield**	**SR**	0.078	22.702	0.151	22.308
	**MLP-GA**	**0.9967**	**1.3319**	**0.9935**	**2.1095**
**BE (%)**	**SR**	0.105	9.146	0.478	6.896
	**MLP-GA**	**0.9775**	**1.4179**	**0.9514**	**2.008**
**TCP**	**SR**	0.091	4.806	0.578	6.207
	**MLP-GA**	**0.87**	**1.9143**	**0.86**	**2.5608**

R^2^; Coefficient of determination, RMSE; Root mean square error.

### Multilayer perceptron-genetics algorithm analysis

**Fig 4:**
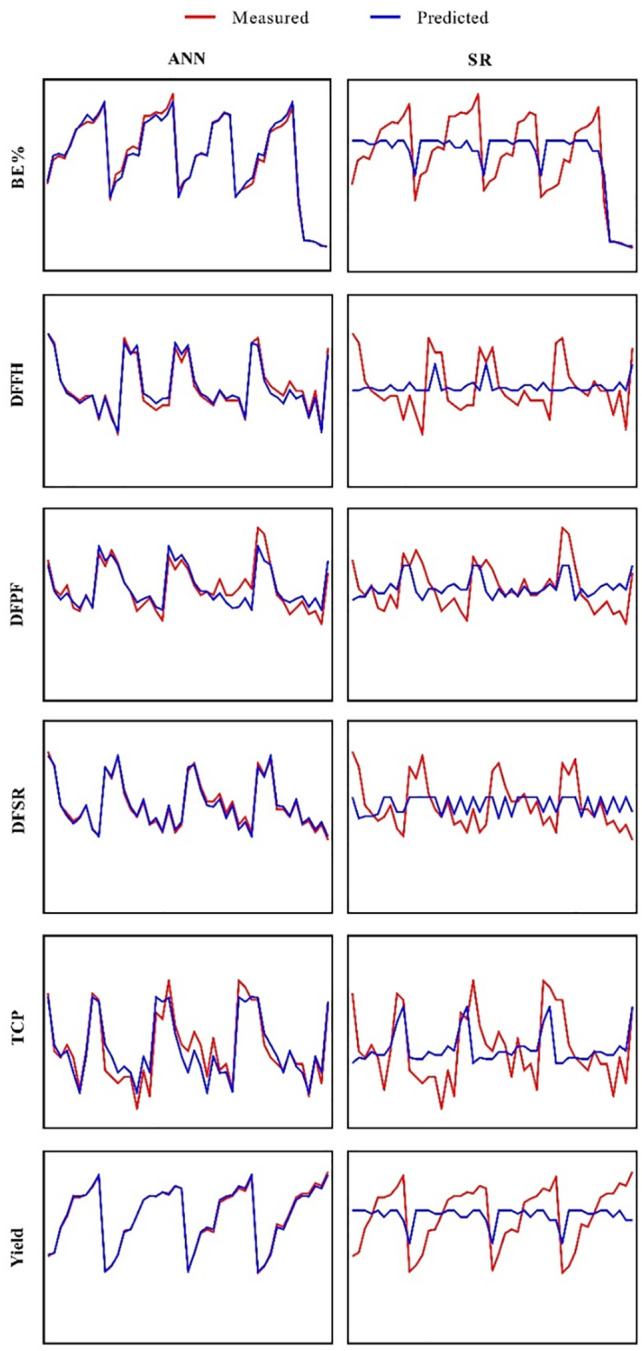
Prediction of yield, biological efficiency (BE), spawn run period (SRP), days for pinhead formation (DPHF), days for the first harvest (DFFH), and total cultivation period (TCP) in *Auricularia cornea* cultivation on different substrates obtained from different ratios of beech and hornbeam sawdust supplemented with wheat and rice brans, based on multilayer perceptron-genetic algorithm (MLP-GA) and stepwise regression (SR) models in training subset.

Initially, BS, HS, WB, and RB concentration levels were applied as inputs and yield, BE, SRP, DPHF, DFFH, and TCP of *A*. *cornea* as outputs. Subsequently, the outputs were forecasted based on developed MLP-GA models. Plotting the forecasted values against the observed values for the training (Fig 4) subset was performed to assess the performance of established MLP-GA models. The high agreement between the forecasted and observed values of yield, BE, SRP, DPHF, DFFH, and TCP of *A*. *cornea* was detected for both training and testing subsets (Table 4). The goodness of fit of established MLP-GA models displayed that they could closely (R^2^ = 0.99, 0.95, 0.97, 0.82, 0.96 and 86) (Table 4) forecast yield, BE, SRP, DPHF, DFFH, and TCP, respectively, of *A*. *cornea* in testing subset, not applied during MLP-GA training processes. Also, established MLP-GA models indicated the balanced statistical values for both training and testing subsets (Table 4). Forecasting the effects of the concentration levels of culture substrate components on yield, NFB, BE, SRP, DPHF, DFFH, and TCP of *A*. *cornea* pave the way for production enhancement and also production cost decrement of this valuable culinary-medicinal mushroom. It is essential to use an accurate modeling system for validly forecasting yield, NFB, BE, SRP, DPHF, DFFH, and TCP of *A*. *cornea* based on the concentration levels of culture substrate components (BS, HS, WB, and RB). This is the first report on developing the mathematical models for forecasting yield, BE, SRP, DPHF, DFFH, and TCP of *A*. *cornea* based on the concentration levels of culture substrate components.

In this research, SR and MLP-GA modeling systems were used to study the relationship between the concentration levels of four culture substrate components “BS, HS, WB, and RB” and the parameters “yield, BE, SRP, DPHF, DFFH, and TCP of *A*. *cornea*”, and thus the possibility of forecasting yield, biological efficiency, spawn run, pinhead formation, first harvest and total cultivation period of *A*. *cornea* using BS, HS, WB, and RB concentration levels. Predictive modeling has not yet been reported for forecasting yield, BE, SRP, DPHF, DFFH, and TCP of *A*. *cornea*.

The results showed that established MLP-GA models could closely forecast yield, BE, SRP, DPHF, DFFH, and TCP of *A*. *cornea* (R^2^ = 0.994, 0.951, 0.969, 0.820, and 0.860, respectively) when meeting unseen data (data of testing subset) (Table 4). Additionally, low hidden neuron numbers (Table 5) as well as the proximity of the error values of testing and training subsets together (Table 4) indicated that overlearning had not occurred in the training process, and established MLP-GA models showed the high generalizability for unseen data [29, 32, 33, 66]. Statistical metrics (RMSE and R^2^) of the training and testing subsets (Table 4) displayed that tansig activation function in the hidden layer was a rational choice for modeling. Small values of RMSE (Table 4) indicated the great performance of established MLP-GA models for forecasting output variables.

**Table 5:** Importance (according to the sensitivity analysis) of the different input variables including different concentration levels of beech (BS) and hornbeam sawdust (HS), and wheat (WB) and rice brans (RB) for achieving maximum yield and biological efficiency (BE), and also minimum spawn run period (SRP), days for pinhead formation (DPHF), days for the first harvest (DFFH), and total cultivation period (TCP) of *Auricularia cornea* using multilayer perceptron-genetics algorithm models (MLP-GA), and also hidden neuron numbers in each developed model.

Criteria	Variable	Importance value (according to VSR^a^)	Neuron number
**Yield**	BS concentration level	0.059	5
	HS concentration level	0.000	
	WB concentration level	1.000	
	RB concentration level	0.497	
**BE (%)**	BS concentration level	0.000	4
	HS concentration level	0.200	
	WB concentration level	1.000	
	RB concentration level	0.092	
**SRP**	BS concentration level	1.000	5
	HS concentration level	0.716	
	WB concentration level	0.000	
	RB concentration level	0.031	
**DPHF**	BS concentration level	1.000	2
	HS concentration level	0.945	
	WB concentration level	0.002	
	RB concentration level	0.000	
**DFFH**	BS concentration level	1.000	4
	HS concentration level	0.997	
	WB concentration level	0.000	
	RB concentration level	0.139	
**TCP**	BS concentration level	0.800	2
	HS concentration level	1.000	
	WB concentration level	0.000	
	RB concentration level	0.070	

^a^ Relative indication of the ratio between the variable sensitivity error and the error of the model when all variables are available. Calculated VSR values were rescaled within range [0, 1].

The yield, BE, SRP, DPHF, DFFH, and TCP of *A*. *cornea* are the complex biological process requiring highly accurate methods for modeling. MLP-GA hybrid model has been successfully applied for modeling the extremely complicated process in different fungal and plant research [32, 35, 67, 68]. A considerable and growing interest in ANN application can be explained by its impressive achievements in problem-solving in a wide variety of research areas, pliable modeling structure, its ability for modeling highly nonlinear and complicated relationships for extra data that make it outperform traditional statistical method, most incredibly regarding the predictive ability [69]. The high predictive accuracy of the training and testing subsets (Table 4) suggested that established MLP-GA could validly forecast yield, BE, SRP, DPHF, DFFH, and TCP of *A*. *cornea*.

### Sensitivity analysis of the models

To classify the inputs in terms of their relative importance on model outputs, VSRs were computed by all data (training and testing subsets). VSRs were estimated for each of the outputs (yield, BE, SRP, DPHF, DFFH, and TCP of *A*. *cornea*) regarding BS, HS, WB, and RB concentration levels (Table 5). Analysis of the yield model displayed that *A*. *cornea* yield was more sensitive to WB concentration level (VSR = 1.000) in culture substrate, followed by RB (VSR = 0.497), BS (VSR = 0.059), and HS (VSR = 0.000) (Table 5). BE displayed more sensitivity to WB (VSR = 1.000), followed by HS (VSR = 0.200), RB (VSR = 0.092), and BS (VSR = 0.000) concentration levels (Table 5). SRP showed more sensitivity to BS (VSR = 1.000), followed by HS (VSR = 0.716), RB (VSR = 0.031), and WB (VSR = 0) concentration levels (Table 5). Accordingly, DPHF exhibited more sensitivity to BS (VSR = 1.000), followed by HS (VSR = 0.945), WB (VSR = 0.002), and RB (VSR = 0.000) concentration levels (Table 5). Also, DFFH displayed more sensitivity to BS (VSR = 1.000), followed by HS (VSR = 0.998), RB (VSR = 0.139), and WB (VSR = 0.000) concentration levels (Table 5). Also, TCP indicated more sensitivity to HS (VSR = 1.000), followed by BS (VSR = 0.800), RB (VSR = 0.070), and WB (VSR = 0.000) concentration levels (Table 5). Notwithstanding the studies on substrate combinations on yield, BE, SRP, DPHF, DFFH, and TCP of *A*. *cornea*, the question had remained open: which substrate displays the most effect on the outputs? As previously stated, sensitivity analysis showed that WB, WB, BS, BS, BS, and HS concentration levels are the most important inputs determining yield, BE, SRP, DPHF, DFFH, and TCP, respectively, of *A*. *cornea* (Table 5).

### Comparison of predictive ability of MLP-GA and stepwise regression models

The statistical values for established MLP-GA models showed so much higher predictive ability than stepwise regression model as computed R^2^ for MLP-GA models vs. stepwise models was: yield = 0.997 vs. 0.078, BE = 0.978 vs. 0.105, SRP = 0.984 vs. 0.130, DPHF = 0.81 vs. 0.21, DFFH = 0.965 vs. 0.064, and TCP = 0.870 vs. 0.091(Table 4 and Fig 4). This higher predictive ability of MLP-GA model than regression models (Table 4 and Fig 4) was likewise described in other research [29, 30, 32, 33].

## Conclusion

MGR, SRP, DFPF, DFFH, NFB, yield, BE, and TCP were studied in this research. Generally, the supplements including WB, RB, and CF added to the BS and HS substrate were useful in terms of the MGR and basidiocarp increment of wood ear mushroom. Overall, WB displayed a high potential for producing native *A*. *cornea* mushrooms. Remarkably, WB is rich in nutrients that can be applied for *A*. *cornea* cultivation. This study used MLP-GA for modeling yield, BE, SRP, DPHF, DFFH, and TCP, respectively, of *A*. *cornea* for the first time. The high accordance between the forecasted and observed values of the output variables (yield, BE, SRP, DPHF, DFFH, and TCP, respectively, of *A*. *cornea*) corroborated the good performance of established MLP-GA models. This work recommended MLP-GA as a strong mathematical tool for forecasting the complex and highly non-linear systems like medicinal mushroom production, *A*. *cornea* production regarding the concentration levels of cultivation substrate components as a case study, therefore, allowing us to present MLP-GA as a powerful modeling method for forecasting in different areas of plant and fungal systems.

## Supporting information

S1 File (DOCX)

## Abbreviations

MGR: Mycelial growth rate
BS: Beech sawdust
HS: Hornbeam sawdust
WB: Wheat bran
RB: Rice bran
BE: Biological efficiency
SRP: Spawn run period
DPHF: Days for pinhead formation
DFFH: Days for the first harvest
TCP: Total cultivation period
SR: Stepwise regression
AI: Artificial intelligence
ANNs: Artificial neural networks
MLP-GA: Multilayer perceptron-genetic algorithm
MEA: Malt extract agar
PDA: Potato dextrose agar
YEA: Yeast extract agar
PEA: Potato extract agar
MEA: Malt extract agar
CRD: Completely randomized design
CF: Corn flour
RCBD: Randomized complete block design
NFB: Number of fruiting body
C/N: Carbon to nitrogen
PCA: Principal component analysis
VSR: Variable sensitivity ratio
R^2^: Coefficient of determination
RMSE: Root mean square error
