# Supplementary material for: Multilayer perceptron-genetic algorithm as a promising tool for modeling cultivation substrate of Auricularia cornea Native to Iran
Source: PLoS One. 2023 Feb 21;18(2):e0281982. doi: 10.1371/journal.pone.0281982 (PMC9942997; doi:10.1371/journal.pone.0281982)
Supplement: S1 File — (DOCX) [file pone.0281982.s001.docx]

**Multilayer perceptron-genetic algorithm as a promising tool for modeling cultivation substrate of *Auricularia cornea* Native to Iran**

**Akbar Jahedi^1^, Mina Salehi^2^, Ebrahim Mohammadi Goltapeh^1^, Naser Safaie^1,^** *

^1^ Department of Plant Pathology, Tarbiat Modares University, Iran

^2^ Department of Plant Genetics and Breeding, Tarbiat Modares University, Iran

*Corresponding author.

*E-mail addresses*: nsafaie@modares.ac.ir

| **Table S1.** GenBank accession numbers of taxa used in the phylogenetic analysis. | | | | |  |  |
| --- | --- | --- | --- | --- | --- | --- |
| **Species Name** | **Sample number** | **Accessions number** | | |  |  |
|  |  | **ITS** |  | **RPB2** |  | |
| *Auricularia africana* | Ryvarden 44929, holotype | MH213350 |  | MZ740061 |  | |
| *A. americana* | Cui 11509 | KT152094 |  | KT152127 |  | |
| *A. angiospermarum* | TJV-93-12-SP | KT152096 |  | KT15212 |  | |
| *A. australiana*, | HN 3213 | MZ647504 |  | ̶ |  | |
| *A. camposii*, | URM 76905 holotype | MH213351 |  | MH213427 |  | |
| *A. asiatica* | OM 13932 | MZ618931 |  | MZ74004 |  | |
| *A. brasiliana* | RSC 359 | KP729276 |  | ̶ |  | |
| *A. conferta* | Dai 18825, holotype | MZ647500 |  | MZ740048 |  | |
| *A. auricula-judae* | Dai 13210 | KM396769 |  | KP729312 |  | |
| *A. cornea* | Dai 15336 | KX022014 |  | KX022074 |  | |
| *A. cornea* | Wu 07 | MH213354 |  | MH213430 |  | |
| *A. cornea* | Cui 11162 | MZ618934 |  | MZ740050 |  | |
| *A. delicata* | P 14, epitype | MH213364 |  | ̶ |  | |
| *A. fibrillifera* | Dai 13598A | KP765615 |  | KX022084 |  | |
| *A. heimuer* | Dai 13765, holotype | KM396793 |  | KP729317 |  | |
| *A. lateralis* | Dai 16417 | MH213369 |  | MH213440 |  | |
| *A. fuscosuccinea* | OM 17909 | KX022029 |  | KX02209 |  | |
| *A. mesenterica* | BRNM 648573 | KP729279 |  | KP729320 |  | |
| *A. minutissima* | Dai 14881, holotype | KT152104 |  | KT152137 |  | |
| *A. novozealandica* | PDD 83897, holotype | KX02203 |  | ̶ |  | |
| *A. nigricans* | Ahti 36234 | KM396802 |  | ̶ |  | |
| *A. nigricans* | TJY-93-242 | KM396803 |  | ̶ |  | |
| *A. orientalis* | Dai 14875, holotype | KP729270 |  | KP72931 |  | |
| *A. pilosa* | LWZ 201904217, holotype | MZ647506 |  | ̶ |  | |
| *A. pusio* | AK 547 | MH213374 |  | ̶ |  | |
| *A. sinodelicata* | Cui 8596 | MH213376 |  | MH213444 |  | |
| *A. subglabra* | Dai 17403 | MH213382 |  | MH213448 |  | |
| *A. scissa* | Ahti 49388 | KM396805 |  | KP72932 |  | |
| *A. srilankensis* | Dai 19575 | MZ647502 |  | MZ740058 |  | |
| *A. submesenterica* | Dai 15450, holotype | MH213386 |  | MH213449 |  | |
| *A. thailandica*, | MFLU 130396 | KR336690 |  | ̶ |  | |
| *A. tibetica* | Dai 15604 | MH213388 |  | MH213453 |  | |
| *A. tremellosa* | Dai 17415 | MH213390 |  | MH213455 |  | |
| *A. villosula* | Dai 13450 | KM396812 |  | KP729327 |  | |
| *Elmerina. efibulata* | Yuan 4525 | MZ618945 |  | MZ740063 |  | |

| **Table S2.** Different cultivation substrates tested for optimizing yield, fruiting body number, biological efficiency, spawn run, pinhead formation, first harvest, and total cultivation period of *Auricularia cornea* | | | | |
| --- | --- | --- | --- | --- |
| **Wheat bran (%)** | **Rice bran (%)** | **Beech sawdust (%)** | **Hornbeam sawdust (%)** | **Substrate** |
| 0 | 0 | 0 | 100 | 1 |
| 0 | 0 | 100 | 0 | 2 |
| 0 | 10 | 0 | 90 | 3 |
| 0 | 10 | 90 | 0 | 4 |
| 10 | 0 | 0 | 90 | 5 |
| 10 | 0 | 90 | 0 | 6 |
| 0 | 20 | 0 | 80 | 7 |
| 0 | 20 | 80 | 0 | 8 |
| 20 | 0 | 0 | 80 | 9 |
| 20 | 0 | 80 | 0 | 10 |
| 0 | 30 | 0 | 70 | 11 |
| 0 | 30 | 70 | 0 | 12 |
| 30 | 0 | 0 | 70 | 13 |
| 30 | 0 | 70 | 0 | 14 |
| 100 | 0 | 0 | 0 | 15 |
| 0 | 100 | 0 | 0 | 16 |


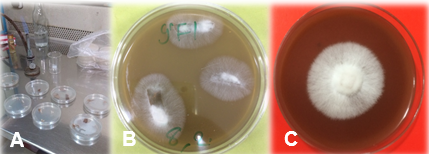


**Fig S1.** Preparation of pure mycelial culture of *Auricularia cornea*. Tissue culture (**A**), mycelium ramification (**B**), pure mycelial culture (**C**)


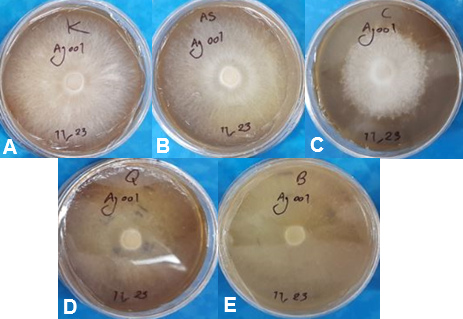


**Fig S2.** Mycellium growth of *Auricularia cornea* on hornbeam extract agar (HAS; **A**), beech extract agar (BEA; **B**), potatao extract agar (PEA; **C**), malt extract agar (MEA; **D**), and yeast extract agar (YEA; **E**) at the best temperature (28 ^o^C)

**
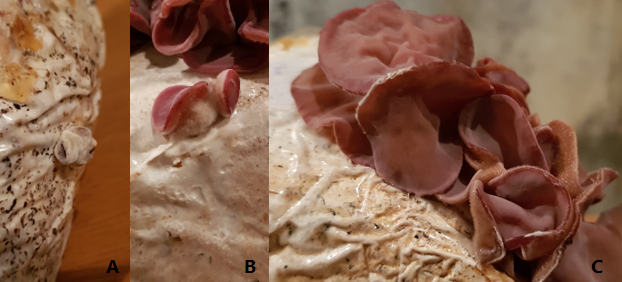
**

**Fig S3.** Different stages of *Auricularia cornea* fruit body development: pinhead formation (A), young fruit body (B) and mature fruit body (C).
